# Stereoselectivity of Interaction of Nonsteroidal Anti-Inflammatory Drug S-Ketoprofen with L/D-Tryptophan in Phospholipid Membranes

**DOI:** 10.3390/membranes12050460

**Published:** 2022-04-24

**Authors:** Anna V. Mastova, Olga Yu. Selyutina, Nikolay E. Polyakov

**Affiliations:** Institute of Chemical Kinetics and Combustion, Institutskaya Str. 3, 630090 Novosibirsk, Russia; mastova-anna99@yandex.ru (A.V.M.); polyakov@kinetics.nsc.ru (N.E.P.)

**Keywords:** ketoprofen, L-tryptophan, D-tryptophan, lipid membranes, electron transfer, stereoselectivity, photolysis, oxidation of lipids, CIDNP, molecular dynamics

## Abstract

The mechanisms of stereoselectivity of the interaction of chiral drugs with active sites of enzymes and cell receptors attract significant attention. The first reason is the difference in therapeutic activity of the enantiomers of the common drugs. Another reason is the interest in the role of chiral inversion of amino acids involved in various peptides in the development of many diseases including Alzheimer’s, Parkinson’s, type II diabetes, and a number of other pathological conditions. In our study we use elementary chemical process—electron transfer (ET) to simulate individual stages of ligand–receptor and enzyme–substrate interactions. In particular, previous studies of photoinduced ET in chiral donor-acceptor dyads consisting of the nonsteroidal anti-inflammatory drug (R/S)-ketoprofen and (L)-tryptophan show the stereo and spin selectivity of ET in diastereomers. The present study is devoted to the interaction of (S)-ketoprofen with L- and D-enantiomers of tryptophan in homogeneous aqueous solution and in phospholipid membranes. The study was done using the NMR technique and molecular modeling. These approaches confirm efficient penetration of ketoprofen into the lipid bilayer and binding with tryptophan molecule. The short-lived paramagnetic intermediates formed during the photoinduced ET from electron donor tryptophan to ketoprofen have been detected using the chemically induced dynamic nuclear polarization (CIDNP) technique. It was found that S-ketoprofen interacts stereoselectively with tryptophan enantiomers in the lipid membrane. The formation of the ketyl radical of ketoprofen under irradiation leads to the oxidation of membrane lipids and may be the cause of ketoprofen phototoxicity. However, in contrast to a homogeneous solution in phosphate buffer saline, where the amino acid tryptophan accelerates the photodecomposition of KP due to intramolecular hydrogen transfer, tryptophan in a lipid membrane significantly reduces the rate of photodegradation due to a reversible electron (or hydrogen) transfer reaction. The stereoselectivity in the rate of KP and lipids decomposition under UV irradiation of S-ketoprofen in the presence of tryptophan enantiomers in lipid bilayer has been detected.

## 1. Introduction

Chiral molecules are the subject of study in many areas of science. Studies of the role of chirality in the origin of life on Earth, as well as practically important searches for the causes of differences in the therapeutic properties of enantiomers of drugs, are still relevant. In recent years, there is an increased interest in studying the mechanisms of spontaneous chiral inversion of amino acid residues in proteins, leading to disruptions in folding processes, which are considered the main cause of Alzheimer’s and Parkinson’s diseases, as well as aging processes. However, the detailed mechanisms of these processes at the molecular level are only assumed and require study using modern physical methods. The establishment of the mechanisms of chiral inversion of amino acids, as well as the nature of the difference in the reactivity and dynamics of peptides containing various optical isomers of amino acid residues, will make it possible to significantly advance in the development of methods for treating many socially significant diseases. The number of stereospecific chemical and biological processes that appeared in the course of pre-biological evolution is constantly growing due to the reactions of new chiral compounds. First of all, these are xenobiotics, drugs, and poisons, as well as herbicides, and various polymeric materials with new useful properties [1]. Chiral compounds interacting with other chiral reagents present both in living systems and in the environment demonstrate stereospecificity. Thus, the rates, and often the direction of the reactions of enantiomers are different. Meanwhile, the different, and often opposite activity of drug enantiomers is a big problem for pharmacology, since most chiral drugs are still used in the form of racemates [2,3,4,5]. Note that stereoselectivity is well established for drug-receptor binding and enzyme catalysis, but has been scarcely investigated for carrier-mediated membrane transport [6,7,8,9].

A similar problem exists in the difference in the properties of proteins and enzymes containing residues of L- and D-amino acids. These proteins are fundamentally different in their dynamic properties. Namely, proteins containing D-amino acid residues are prone to aggregation, leading to changes in conformational dynamics and the association of internally disordered amyloid proteins (failures in the so-called protein folding). These failures, as it was mentioned above, are considered one of the causes of Alzheimer’s, Parkinson’s, and other diseases [10]. According to modern concepts, the amyloid-β (Aβ) pathway is positioned at the center of Alzheimer’s disease pathophysiology [11]. Aβ is generated as a soluble monomer and in physiological conditions, it is involved in neuronal cytoprotective pathways and intracellular signaling. However, it could self-assemble forming dimers, oligomers, protofibrils, and fibrils. Fibrils can accumulate in plaques, typically viewed as an Alzheimer’s disease neuropathological hallmark [11]. The molecular dynamics underlying the incipient Aβ monomer self-assembly are still under debate, but there are several hypotheses explaining this process. For example, less soluble Aβ demonstrated a higher ability to aggregate. Glycation could affect the solubility of Aβ and, as a result, the ability of Aβ to form self-associates [12]. Another hypothesis explaining the self-association of Aβ is connected with the accumulation of D-amino acids in the Aβ structure [13]. Although, D-amino acids are studied as potential therapeutic agents against Alzheimer’s disease [14,15]. All D-peptides are considered potential agents breaking the self-association of L-rich Aβ [16].

Another possible reason for these pathologies is the action of free radicals (toxic reactive oxygen species, etc.) [17,18]. Some authors [1] suggest a possible connection between these two factors, namely, the radical mechanism of the processes leading to changes in the conformational dynamics of amyloid proteins. The discussed process is a spontaneous change in the optical configuration (spontaneous chiral inversion, SCI) in peptides [19]. In this case, we mean a change in the optical configuration of amino acid residues from L- to D-, which leads, as it is known, to pathological processes. Such a chiral inversion is called spontaneous and occurs under the action of enzymes and transport proteins [19]. In connection with the aforementioned practical significance, the effect of SCI of amino acids on the structure and conformational mobility of peptides is being intensively studied both by experimental methods and by molecular modeling [3,17,18,19,20,21,22,23,24].

Certain progress in this direction in the last decade was achieved by the study of model elementary processes, including electron and energy transfer, in linked systems with two chiral centers [20,21,22,23,24,25,26,27,28,29,30]. Dyads in which a chiral drug molecule (electron acceptor) is covalently bonded to a chiral amino acid (electron donor) have been considered as models for the binding of a drug molecule to an amino acid residue in the enzyme active site. For example, studies of dyads involving naproxen linked with (S)-tryptophan demonstrate stereo selectivity of electron transfer. The stereoselectivity in the rate of photoinduced electron transfer and lifetime of excited states has been detected also for enantiomers of ketoprofen—(S)-tryptophan dyad [30].

The present study is devoted to the role of the optical configuration of amino acid tryptophan in its reactivity in a phospholipid membrane. Note that membrane lipids and amino acids are almost ubiquitously homochiral within individual cells on Earth. A number of studies try to elucidate the determinants of stereospecific interactions between lipids, amino acids, and chiral drug molecules (see for example review [31]). Taking into account the results of previous studies of photoinduced electron transfer in diastereomers ketoprofen-tryptophan, in the present study we also use S-ketoprofen as an electron acceptor in photoinduced interaction with enantiomers of tryptophan, and CIDNP technique (chemically induced dynamic nuclear polarization) and molecular dynamics (MD) to study the peculiarities of their interaction in lipid membranes. In addition, the effect of ketoprofen–tryptophan interaction on free radical-mediated lipid peroxidation has been studied.

Ketoprofen (2-(3-benzoylphenyl)propionic acid, KP) is a nonsteroidal anti-inflammatory drug (NSAID) with analgesic and antipyretic effects. KP reversibly inhibits cyclooxygenase-1 and -2 (COX-1 and COX-2) enzymes, which regulate the production of proinflammatory prostaglandin precursors. Although the anti-inflammatory role of two KP enantiomers is not fully characterized, it is known that R-KP is a weak cyclooxygenase inhibitor, being 100 to 1000 times less potent than the S-enantiomer in vitro. In addition, ketoprofen is known to be high photosensitive and may induce phototoxic and photoallergic reactions [32,33,34,35,36,37].

**Figure 1:**
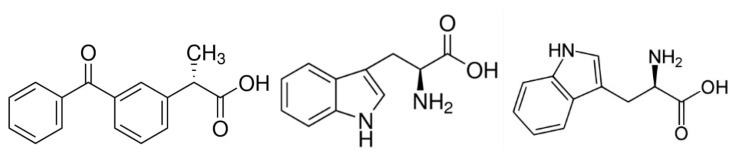
Chemical structures of S-Ketoprofen, L-Tryptophan, and D-Tryptophan optical isomers.

Due to its benzophenone-like structure (Figure 1), KP can participate in various photo-redox processes resulting in the formation of toxic free radicals and potentially allergic photoproducts. The photosensitization mechanism of KP under UV irradiation is extensively studied by many researchers [38,39,40,41,42,43,44]. In particular, the application of CIDNP and NMR techniques allowed the establishment of new radical mechanisms of ketoprofen phototransformation in a homogeneous solution [42]. It includes the breaking of the C–C bond with the formation of the neutral triplet radical pair of benzyl and ^●^COOH radicals. In addition, it was shown that in the presence of hydrogen donors and an additional radical reaction channel, namely, photoreduction with the formation of a ketyl radical, can occur. The ratio between these reaction channels substantially depends on the nature of the environment and the presence in the system of a suitable electron or hydrogen donor. However, various aspects of the photoinduced interaction of KP with biological molecules are still under debate. In particular, it was demonstrated that amino acids tryptophan, tyrosine, and histidine can accelerate the reaction to produce a KP biradical in the phosphate buffer solution (pH 7.4) [45,46,47].

Taking into account the significance of free radicals in the mechanism of ketoprofen phototoxicity, and a possible important role of amino acid residues in ketoprofen phototransformation, in the present work we have studied the photolysis of (S)-ketoprofen with enantiomers of tryptophan in homogeneous buffer solutions and in phospholipid membranes that mimic the biological environment. The binding between ketoprofen and tryptophan was studied by NMR technique and MD simulation. The short-lived paramagnetic intermediates formed during the photoinduced electron transfer from electron donor tryptophan to ketoprofen were detected using CIDNP technique. The mechanism of stereoselectivity of the degree of lipid decomposition under UV irradiation of S-ketoprofen in the presence of tryptophan enantiomers was also discussed on the basis of NMR data and molecular dynamics modeling.

## 2. Materials and Methods

### 2.1. Materials

Ketoprofen (98%, Sigma, KP, St. Louis, MO, USA), L-tryptophan (L-Trp), D-tryptophan (D-Trp) (98%, Aldrich, St. Louis, MO, USA), and deuterated solvent D_2_O (99.9% D) were used as received.

**Figure 2:**
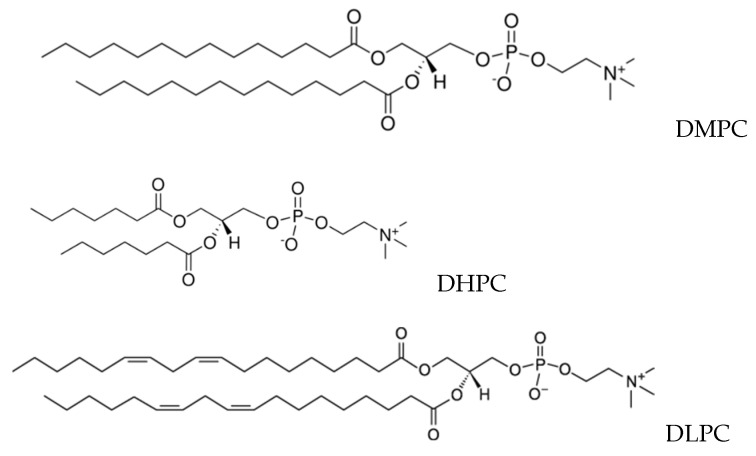
Structures of lipids DMPC (1,2-dimyristoyl-sn-glycero-3-phosphocholine), DHPC (1,2-diheptanoyl-sn-glycero-3-phosphocholine and DLPC (1,2-dilinoleoyl-sn-glycero-3-phosphocholine).

Bicelles were formed from DMPC (1,2-dimyristoyl-sn-glycero-3-phosphocholine) or DLPC (1,2-dilinoleoyl-sn-glycero-3-phosphocholine) and DHPC (1,2-diheptanoyl-sn-glycero-3-phosphocholine, Avanti Polar Lipids, purity > 99%, Alabaster, AL, USA, Figure 2). Powder components were pre-dissolved in chloroform. After solvent evaporation, the dry lipid film was hydrated with D_2_O. The final concentration of lipid was 24 mM. To accelerate the formation of bicelles, three freeze-thaw cycles were performed. The DMPC:DHPC and DLPC:DHPC ratios were 1:2.

### 2.2. NMR Study

^1^H NMR spectra were measured on Bruker Avance HD III NMR spectrometer (500 MHz ^1^H operating frequency, Bruker, Billerica, MA, USA). CIDNP experiments were performed on Bruker DPX-200 NMR spectrometer (200 MHz ^1^H operating frequency). Lambda Physik EMG 101 MSC excimer laser was used as a light source (308 nm, 100 mJ at output window, 20 mJ/pulse in sample volume, pulse duration 15 ns) in the CIDNP experiments. The samples were irradiated in standard 5 mm Pyrex NMR tubes directly in the probe of NMR spectrometer. To enhance the signal-to-noise ratio in the present study we used the pseudo-steady-state (PSS) photo-CIDNP method. The PSS experiments were performed using standard pulse sequence: presaturation–delay 1–P1(π)–delay 2 (16 laser pulses with repetition rate 50 Hz during delay 2)–observation P2(π/2)–acquisition.

### 2.3. Molecular Dynamics Simulations

Molecular dynamics simulations were performed to understand the interactions of KP with phospholipid-containing membranes and Trp molecules using the GROMACS 2018.4 package and GROMOS54a7 force field. The topologies of KP and Trp were built using the Automated Topology Builder [48]. For lipid simulations, the model lipid DMPC (1,2-dimyristoyl-sn-glycero-3-phosphocholine) introduced by Poger and Mark was utilized [49]. The simple point charge (SPC) model of water molecules was used.

The simulation was performed in the NPT ensemble with constant pressure (1 bar) and constant temperature T = 310 K, which were maintained by the semi-isotropic Parrinello–Rahman barostat [50] and Nose–Hoover thermostat [51]. For electrostatic interactions, the PME method with the fourth-order of cubic interpolation and a grid of 0.16 was used [52]. The initial configuration of the system contained the bilayer consisting of 128 lipid molecules surrounded by water (~10,000 water molecules) and KP and Trp molecules located in water outside the bilayer. For all systems with KP and Trp, one production run of 500 ns duration was performed.

## 3. Results

As a model of drug interaction with the active sites of cellular receptors and enzymes, the photoinduced interaction of ketoprofen (KP) with the enantiomers of the amino acid tryptophan (Trp) was studied in this work. Of particular interest is to study these processes in model lipid membranes (bicelles), since the results can shed light on both widely discussed problems, namely the stereoselectivity of drugs activity and the mechanism of KP phototoxicity.

### 3.1. NMR Study

**Figure 3:**
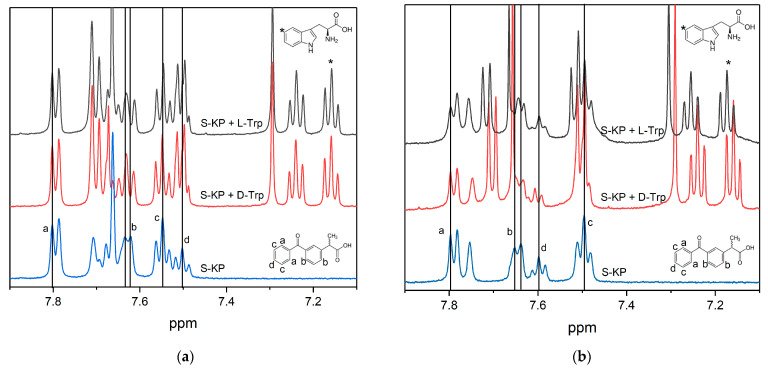
^1^H NMR spectra of S-KP in the absence (blue) and in the presence (black and red) of L/D-Trp in bicelles (**a**) in PBS at pH = 7.4; (**b**) at pH = 4.

In contrast to a pure solution in phosphate buffer (PBS), the solubility of KP in the bicelle solution is much higher, which confirms the inclusion of KP in the bicelle. Some peculiarities of the inclusion of KP and Trp molecules into lipid membrane have been studied earlier by NMR and MD simulation techniques [53]. In particular, the NMR selective NOESY technique was applied to study the interactions between KP and Trp. The presence of cross-peaks of Trp and KP with lipid protons indicates the incorporation of Trp and KP molecules into the lipid bilayer. MD simulation has shown a significant influence of Trp molecules on the position of KP inside the bilayer [53]. Upon addition of Trp, a slight shift of KP NMR lines is also observed in the spectrum, which indicates KP–Trp interaction within the bilayer (Figure 3).

The most significant differences are observed for protons of KP marked with symbol (b) (Figure 3). The shift of this proton is higher in the case of deprotonated KP state (pKa value is 4.7) at pH = 7.4 than in the case of protonated KP state at pH = 4. It could mean a stronger interaction of KP with Trp in the case of a deprotonated KP state.

### 3.2. KP Photolysis in Phospholipid Bicelles in the Presence of L/D-Tryptophans

**Figure 4:**
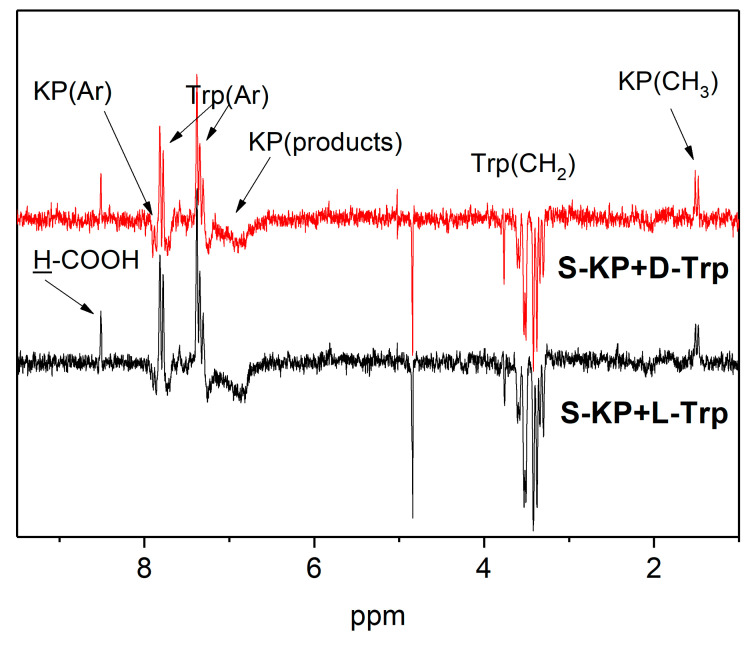
CIDNP spectra detected during photolysis of 2 mM KP in the presence of 4 mM L- and D-tryptophan in PBS at pH 7.4.

The mechanism of KP photolysis in the presence of amino acid tryptophan in homogeneous solutions, in phospholipid membranes, and in covalently linked donor-acceptor dyads has been described in detail in our previous studies [25,30,42,53]. The short-lived paramagnetic intermediates formed during the photoinduced ET from electron donor tryptophan to ketoprofen have been detected in these works using the chemically induced dynamic nuclear polarization (CIDNP) technique. CIDNP phenomenon (non-equilibrium distribution of the intensities of NMR signals of reaction products formed in radical chemical reactions) is a powerful technique for studying the mechanisms of photoinduced chemical processes and the structures of paramagnetic reaction intermediates [54]. CIDNP effects are formed in the pair of two radicals formed via breaking the chemical bond or via electron or hydrogen atom transfer between the corresponding donor and acceptor. This is why the observation of CIDNP effects in a chemical reaction is direct evidence of the involvement of free radicals in the reaction scheme [55]. Moreover, taking into account that the CIDNP intensity is proportional to the values of hyperfine interaction constants in the radical–precursor of polarized product, we can consider the CIDNP spectrum as a “portrait” of free radical intermediates participating in this reaction [56]. As an example, Figure 4 shows the CIDNP effects detected during photolysis of KP in PBS in the presence of Trp isomers.

As one can see from Figure 4, no difference in CIDNP effects for L/D-tryptophans has been detected in a homogeneous PBS solution. On the other hand, strong stereoselectivity of CIDNP intensity had been reported for covalently linked KP-Trp dyads [25]. The analysis of CIDNP sign of Trp protons (enhanced absorption on aromatic protons and emission on CH_2_ protons) using Kaptein rules [54] leads to the conclusion that this polarization was formed in triplet radical pair of Trp radical (a(indol) < 0, a(CH2) > 0, Δg < 0) and ketyl radical via reversible electron (or hydrogen atom) transfer from Trp molecule to KP in triplet excited state. Additionally, one can see the CIDNP effects on methyl protons of KP (1.5 ppm) and reaction product—formic acid HCOOH (8.5 ppm). These effects are formed in parallel reaction channels, namely in monomolecular C–C bond breaking in KP triplet excited state with formation of radical pair of ^●^COOH and benzyl radicals [42].

**Figure 5:**
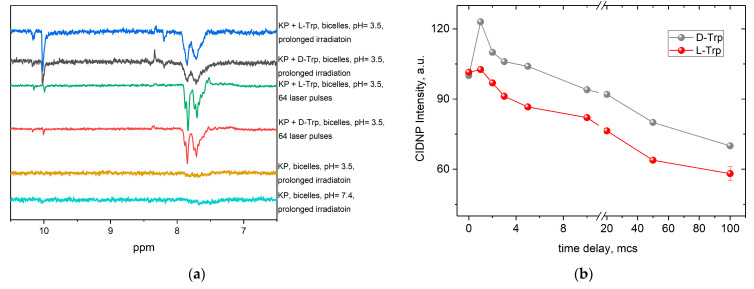
CIDNP spectra detected during photolysis of 2 mM S-KP in the absence and in the presence of 4 mM L/D-Trp in DHPC/DMPC bicelles at pH = 3.5 at pH = 7.4 (**a**); kinetics of TR CIDNP intensity of KP protons (7.8 ppm) at pH 3.5 (**b**).

When the photolysis of KP in the presence of Trp occurs in the lipid bilayer, the CIDNP effects have been observed on the reaction products and aromatic protons of KP (Figure 5). The main difference in the mechanisms of KP photolysis in homogeneous aqueous solution and in lipid bilayer occurs from the presence of water molecules which accelerate intramolecular hydrogen transfer followed by elimination of CO_2_ and formation of biradical [41]. The reversible electron transfer reaction between triplet KP and Trp is a minor process in a homogeneous solution. On the other hand, in the lipid membrane, the contribution of intramolecular hydrogen transfer decreases significantly due to the absence of water, and the reversible electron (or hydrogen) transfer reaction between triplet KP and Trp becomes the main reaction channel.

Note that light in this reaction is predominantly absorbed by KP molecules, whose extinction coefficient is an order of magnitude higher than that for Trp [53]. It can be seen that polarized reaction products appear in the region of 8–10 ppm, which is typical for lipid oxidation products [57]. In addition, the polarization of aromatic protons of KP was observed at 7.5–7.8 ppm. This polarization was not observed in the absence of Trp, so we can conclude that it formed in radical pair of ketyl and Trp radicals. The analysis of the CIDNP sign of KP protons (emission on aromatic protons) using Kaptein rules leads to the conclusion that this polarization was formed in triplet radical pair of ketyl radical (a(o,p) < 0, Δg > 0) and Trp radical via reversible electron (or hydrogen atom) transfer.

Note that the CIDNP intensity is higher for the L-enantiomer of Trp (Figure 5a). This result is in accordance with previously studied diastereomers of covalently linked KP-Trp dyads. As was demonstrated by Ageeva A.A. with coauthors [25], (S,S)-KP-Trp dyad exhibits much stronger CIDNP effects compared with (S,R) and (R,S) diastereomers. This means that weak non-covalent interactions between chiral molecules can also be responsible for the stereoselective effects of the drug’s enantiomers.

We assume that polarization on lipid oxidation products near 10 ppm is formed in secondary processes during the photoinduced interaction of these reaction products with Trp molecules. This suggestion is confirmed by the observed increase in the CIDNP intensity of these products during photolysis with simultaneous decrease in KP concentration. This result shows that the phototoxicity of KP can be caused not only by KP-derived radicals but also by reactions of photosensitive products with lipids and embedded peptides.

Time-resolved CIDNP experiments (Figure 5b) also show the difference in kinetics of CIDNP formation for L and D-Trp. The time course of polarization intensity of KP protons is typical for recombination products of radical pairs with Trp radical [56,58].

**Figure 6:**
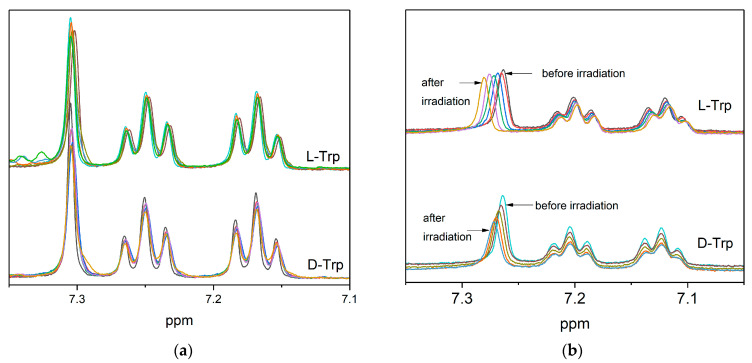
^1^H NMR spectra before and after irradiation (wavelength of the laser light is 308 nm) of 4 mM L/D-Trp in the presence of 2 mM S-KP in DHPC/DMPC bicelles in PBS at pH = 7.4 (**a**); at pH = 3.8 (**b**) showing the chemical shift of the singlet and decomposition of the Trp.

**Figure 7:**
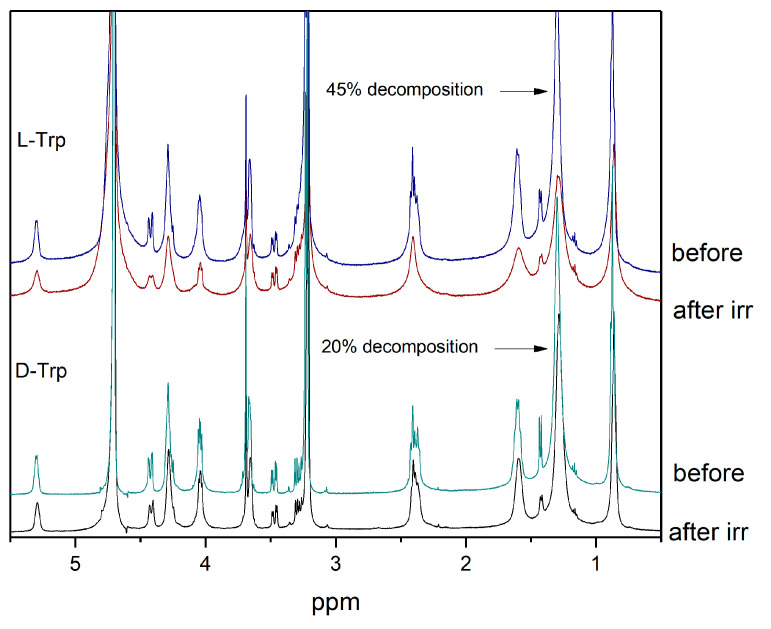
CIDNP spectra detected during photolysis of 2 mM KP in the presence of 4 mM L- and D-tryptophan in PBS at pH 7.4.

It should be noted also that in the absence of tryptophan, the photodegradation of KP is much faster than in the presence, and accompanied by a decrease in the intensity of lipid signals (approximately 30% after 900 laser pulses), which may be a consequence of radical lipid polymerization. At the same time, in the presence of tryptophan, the decrease in the intensity of lipid signals is lower, which indicates the reversibility of the reaction of KP with tryptophan in the lipid bilayer. The high efficiency of quenching the excited state of KP with tryptophan may be associated with their close interaction in the ground state in the lipid bilayer, as it follows from NMR experiments and molecular modeling. NMR experiments show a clear difference in the behavior of L/D-Trp NMR signals at pH 7.4 (PBS buffer) and pH 3.8 (Figure 6). This is an additional confirmation of the difference in the interaction of KP with Trp isomers in the lipid bilayer. It is important that there is also a difference in the degree of decomposition of lipids and KP molecules after irradiation: in the oxygenated system with L-tryptophan, the integral intensity of the peak corresponding to the CH_2_ groups of lipids decreased by 45%, but in the system with D-tryptophan by 20% (Figure 7).

**Figure 8:**
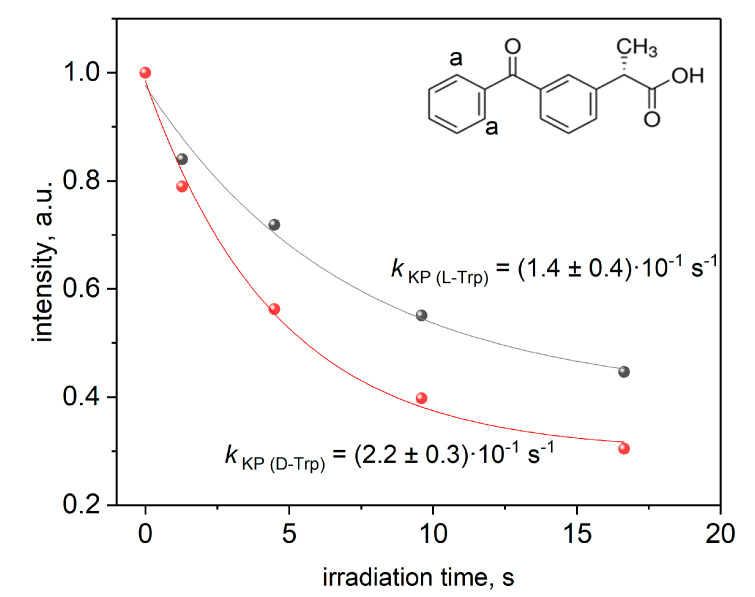
Rate constants of the decomposition of KP and L/D-Trp under irradiation in DHPC/DMPC bicelles.

The noticeable difference in the decomposition rate of KP and Trp molecules during the photolysis was also observed only in acidic media where KP is in protonated form. The dependence of the intensity of KP ((a)-protons, Figure 3) and Trp signals ((*)-protons, Figure 3) on irradiation time was measured. The example of decay kinetics is given in Figure 8. The rate constants are given in Table 1.

**Figure 9:**
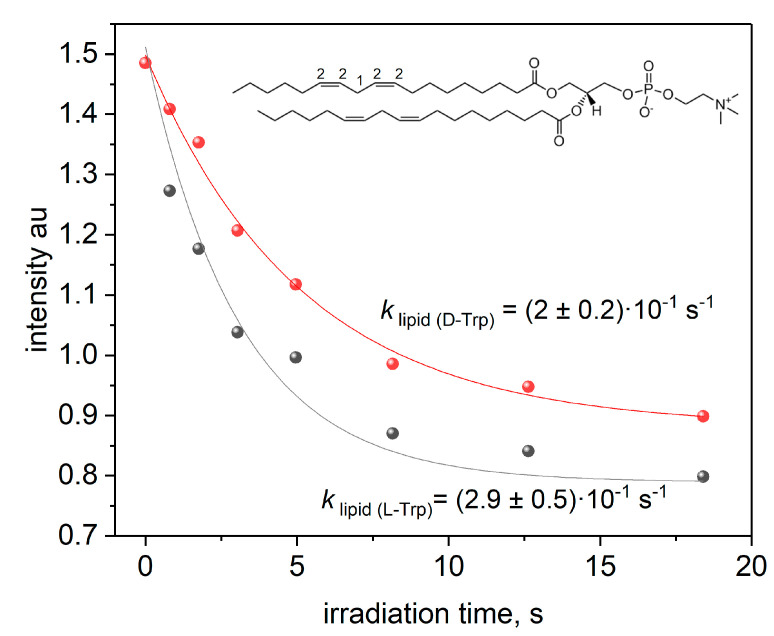
Decay kinetics of ^1^H NMR signals lipid protons (1) at 2.7 ppm on irradiation time in DHPC/DLPC bicelles at pH = 3.3.

All the results shown above were obtained in bicelles consisting of saturated lipids. In addition, we have studied the lipids oxidation in bicelles consisting of unsaturated lipids DHPC/DLPC (see Figure 1) at pH = 3.3 in O_2_ saturated solution (Figure 9). The analysis of the kinetic of unsaturated lipids oxidation was done with the approach described in our previous studies [59,60,61]. Briefly, the time dependence of the decay of the NMR signal of bis-allylic protons of lipid (1-H, Figure 9) was measured. Since the initiation stage of unsaturated lipid oxidation is the abstraction of a hydrogen atom at this position [62], and reaction products (lipid radicals and conjugated dienes) do not contain such protons in the structure, the initiation stage leads to a decrease in the intensity of this signal as a function of time. Similar decay kinetics have been observed for NMR signals of protons 2-H (non-conjugated double bonds).

The rate constants are given in Table 2.

One can see a noticeable difference in the initiation rates of lipids oxidation induced by KP photolysis in the presence of L/D-Trp optical isomers.

### 3.3. MD Simulation Study

**Figure 10:**
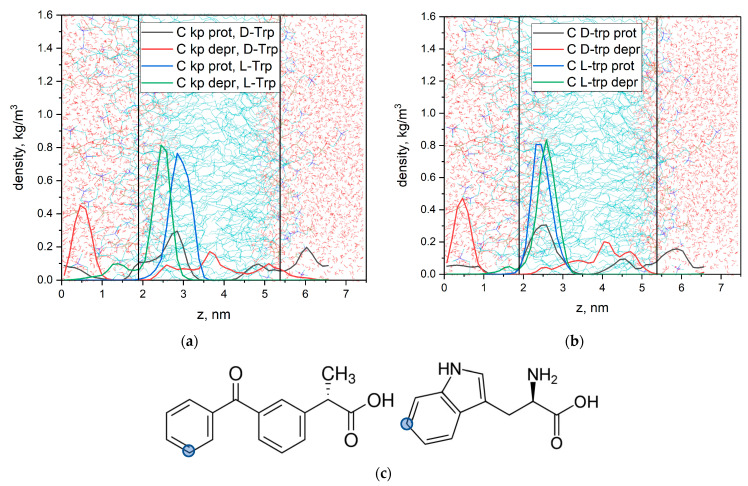
Density profiles of the selected C atoms of KP (**a**), L/D-tryptophans (**b**) in lipid bilayer for protonated (prot) and deprotonated (depr) states of KP and atom selections (**c**). Vertical lines correspond to the centers of density profiles of DMPC N atoms.

To better understand the nature of observed differences in the interaction of KP with L/D-tryptophan MD simulations of KP and L/D-tryptophan in lipid bilayer were done. Figure 9 shows calculated density profiles of the selected C atoms (see Figure 10c) across the box. The lipid bilayer is centered at the center of the box.

As was shown in our previous study, deprotonated KP molecules quickly (~20 ns) penetrate into the lipid bilayer, but in contrast to protonated molecules, they could leave the hydrophobic region, remaining bound to the bilayer surface. These results are in good agreement with the results obtained by Okazaki et al. [63], which indicate that the KP molecule penetrates into the lipid bilayer.

It is noticeable, that the localization significantly differs for L- and D-Trp. L-Trp is located predominantly inside the bilayer, while D-Trp could leave the bilayer more freely. Therefore, D-tryptophan demonstrated less affinity to the lipid membrane. The same difference in KP behavior in the presence of L/D-Trp is observed. Free exchange of KP and Trp molecules through bilayer could explain the slower reaction rate of lipid decomposition in the case of D-Trp. These results correlate with data on the kinetics of decomposition of lipids in the presence of KP and L/D-Trp. Due to the deeper insertion of KP and L-Trp in the hydrophobic part of the membrane, the reaction with lipid could be faster than in the case of D-Trp.

**Figure 11:**
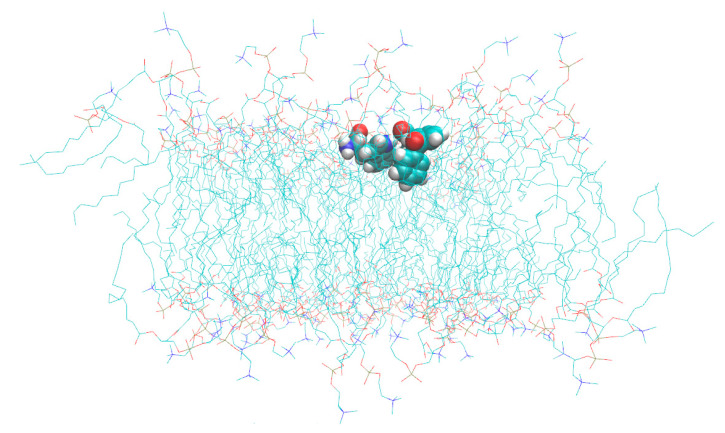
Snapshot of MD trajectory with deprotonated KP-D-Trp associate. Water molecules are not shown.

We have also calculated mean distances between selected C atoms of KP and Trp (Figure 10c) from MD trajectories. For the protonated form of KP mean distance is 2.5 ± 0.4 nm, and for the deprotonated form mean distance is 1.2 ± 0.3 nm. Therefore, in the case of deprotonated KP molecule, the distance between aromatic protons of KP and Trp is two times lower than in the case of protonated KP molecule. Moreover, the association of deprotonated KP with D-Trp was observed (Figure 11). This association remained stable during ~100 ns (20% of production run duration). Carboxyl group of KP molecule interacts with aromatic NH-group of Trp. This fact could explain the significant shift of KP protons in the presence of Trp in bicelles under pH = 7.4 when KP is in a deprotonated state (Figure 3a). The most significant shift is observed for (b)-protons of KP, which are located near the carboxyl group.

Note that we do not observe stable associates of KP-L-Trp during the simulation time (500 ns).

## 4. Conclusions

A big part of drugs in the market belongs to chiral compounds which demonstrate a significant difference in the therapeutic activity of chiral isomers. Since the physicochemical properties of chiral isomers are identical in solutions, we assume that the difference in the therapeutic activity arises in the interaction of chiral drugs with other chiral molecules in living systems, for example, with amino acid residues located in the active sites of enzymes and cell receptors. In our study we use elementary chemical process—electron transfer (ET) to model individual stages of ligand–receptor and enzyme–substrate interactions. Another reason for our interest in these model processes is the elucidation of the role of chiral inversion of amino acids involved in various peptides in the development of many diseases including Alzheimer’s, Parkinson’s, and a number of other pathological conditions. For this purpose, we have studied the photoinduced interaction of nonsteroidal anti-inflammatory drug ketoprofen with L- and D-isomers of tryptophan in solution and in a model phospholipid membrane. KP effectively penetrates into the lipid membrane and reacts with lipids by hydrogen abstraction, and it leads to the damage of the membrane. Other targets of triplet KP are the peptides located inside the cell membrane. It was demonstrated that in the presence of amino acid tryptophan, the quenching of KP excited state by Trp via electron transfer occurs more effectively than the reaction with lipids. In contrast to a homogeneous phosphate buffer solution, where the amino acid tryptophan accelerates the photodecomposition of KP due to intramolecular hydrogen transfer [45,46,47], tryptophan in a lipid membrane significantly reduces the rate of photodegradation due to a reversible electron (or hydrogen) transfer reaction. The main difference in the mechanisms of KP photolysis in homogeneous aqueous solution and in the lipid bilayer occurs from the presence of water molecules which accelerate intramolecular hydrogen transfer followed by the elimination of CO_2_ and formation of biradical. The reaction of biradical with amino acids results in the formation of free radicals and the irreversible decay of KP. The reversible electron transfer reaction between triplet KP and Trp is a minor process in a homogeneous solution. On the other hand, in the lipid membrane, the contribution of intramolecular hydrogen transfer decreases, and the reversible electron transfer reaction between triplet KP and Trp becomes the main reaction channel.

In the present study, we have demonstrated that the binding between S-ketoprofen with L- and D-enantiomers of tryptophan in phospholipid membranes results in stereoselectivity of the photoinduced radical processes. The stereoselectivity in the rate of KP and lipids decomposition under UV irradiation of S-ketoprofen in the presence of tryptophan enantiomers has been detected. The results of MD simulations show that the stereoselectivity of KP-Trp interaction also appears in the interaction of these molecules with the lipid bilayer. It agrees with modern data on the role of chirality in phospholipid membranes’ interaction with chiral molecules. Chiral lipid membranes can discriminate between amino acid enantiomers. A natural cellular membrane interacts more favorably with the L-enantiomer than the D amino acid [31]. Using a computer simulation, Chen et al. have shown that L-peptides are absorbed easily by natural POPC membranes, but the D-peptides have to overcome an extra free-energy barrier [64]. This finding was confirmed experimentally by T. Ishigami et al. [65] and Hu et al. [66] who demonstrated chiral separation of amino acids by model lipid membranes. They found that L-amino acids permeated into lipid bilayer much faster than D-amino acids. The authors suggested that hydrogen bonds drive amino acid adsorption into lipid membranes and that the stereospecificity of lipid membranes is a result of stereospecific hydrogen bonds formation with side chains of amino acid. Overall, one can conclude that amino acids tend to preferentially interact stronger with lipids of the same L/D chirality. Similarly, drug molecules tend to preferentially interact stronger with amino acids of the same chirality. The nature of the stereoselectivity of the interaction of amino acid with lipid membrane is still under debate, but obtained results demonstrated one more example of this effect.

Obtained results are in agreement with our previous study of the dyads in which L- and D-Trp were covalently bounded to S-ketoprofen: the efficiency of photoinduced electron transfer in homogeneous acetonitrile solution was much higher in a dyad with L-isomer of Trp [25]. As for (R,S)- and (S,R)-configurations of dyads, they had completely identical NMR spectra and demonstrate identical efficiency of electron transfer. These results allow us to suggest that it is the mutual influence of the two chiral centers that most likely determines the differences in the therapeutic effect of drug enantiomers, as well as in the side reactions of chiral drugs. Considering that a change in the configuration of amino acids can lead to a change in the secondary and tertiary structure of proteins, we can expect even greater differences in the strength of the interaction and in the rate of reaction of ketoprofen with peptides containing various tryptophan isomers.

## Figures and Tables

**Table 1:** Rate constants of the decomposition of KP and L/D-Trp under irradiation in DHPC/DMPC bicelles.

	Rate Constant (×10^−1^ s^−1^)
L-Trp decomposition, KP + Trp, pH = 4	0.2 ± 0.05
D-Trp decomposition, KP + Trp, pH = 4	0.7 ± 0.01
KP decomposition, KP + L-Trp, pH = 7.4	3 ± 0.2
KP decomposition, KP + L-Trp, pH = 4	1.4 ± 0.4
KP decomposition, KP + D-Trp, pH = 7.4	3 ± 0.2
KP decomposition, KP + D-Trp, pH = 4	2.2 ± 0.3

**Table 2:** Rate constants of the decay of 1-H and 2-H lipid signals under irradiation in DHPC/DLPC bicelles with KP and L/D-Trp, pH = 3.3.

	Rate Constant (×10^−1^ s^−1^)
1-H decay, KP + L-Trp	2.9 ± 0.5
1-H decay, KP + D-Trp	2 ± 0.2
2-H decay, KP + L-Trp	2.5 ± 0.3
2-H decay, KP + D-Trp	1.4 ± 0.1

## Data Availability

Data is provided in the article.
